# Comparison of Survival Between Primary Debulking Surgery Versus Neoadjuvant Chemotherapy for Ovarian Cancers in a Personalized Treatment Cohort

**DOI:** 10.3389/fonc.2020.632195

**Published:** 2021-02-10

**Authors:** Zheng Feng, Hao Wen, Ruimin Li, Shuai Liu, Yi Fu, Xiaojun Chen, Rui Bi, Xingzhu Ju, Xiaohua Wu

**Affiliations:** ^1^ Department of Gynecological Oncology, Fudan University Shanghai Cancer Center, Shanghai, China; ^2^ Department of Oncology, Shanghai Medical College, Fudan University, Shanghai, China; ^3^ Department of Radiology, Fudan University Shanghai Cancer Center, Shanghai, China; ^4^ Department of Nuclear Medicine, Fudan University Shanghai Cancer Center, Shanghai, China; ^5^ Center for Biomedical Imaging, Fudan University, Shanghai, China; ^6^ Shanghai Engineering Research Center of Molecular Imaging Probes, Fudan University, Shanghai, China; ^7^ Department of Pathology, Fudan University Shanghai Cancer Center, Shanghai, China

**Keywords:** ovarian cancer, primary debulking surgery, neoadjuvant chemotherapy, residual disease, progression-free survival, overall survival

## Abstract

**Objective:**

To compare survival between primary debulking surgery (PDS) and neoadjuvant chemotherapy (NACT) for the treatment of ovarian cancer patients per our selective protocol.

**Methods:**

Between Sep 1^st^, 2015, and Aug 31^st^, 2017, 161 patients were enrolled in our prospective cohort. All of the patients received preoperative clinic-radiological assessments, according to the Suidan criteria for R0 resection. Patients with a score of 0–2 received PDS. Patients with a score of ≥3 were counseled on the choices of PDS, NACT, or an optional staging laparoscopy, according to the Fagotti criteria. Clinic-pathological data were prospectively collected until May 1^st^, 2020, and the impacts of different treatment strategies on progression-free survival (PFS) and overall survival (OS) were analyzed.

**Results:**

110 patients underwent PDS, and 51 patients received NACT with consequent interval debulking surgery. The R0 resection rate was 57.8%. All but one of the patients received platinum-based chemotherapy, and 105 (65.2%) patients were platinum-sensitive. Based on the univariate analysis, the PDS group exhibited prolonged PFS compared with the NACT group (P=0.029). The subgroup analysis showed that patients receiving NACT with residual disease (RD) exhibited the worst PFS (P=0.001). Based on the multivariate analysis, NACT with RD was still an independent impaired factor for PFS (P=0.04). However, NACT did not affect OS in the univariate or multivariate analyses.

**Conclusion:**

In our prospective cohort, NACT ovarian patients exhibited inferior PFS and noninferior OS compared with PDS patients. Given our selective protocol, NACT cannot be arbitrarily denied while appropriate PDS is still a priority.

## Introduction

Ovarian cancer (OC) continues to be the most lethal disease among females worldwide (1), and the five-year overall survival rate is lower than 50% (2). Primary debulking surgery (PDS) and individual platinum-based adjuvant chemotherapy are the standard treatments for ovarian cancer patients. However, neoadjuvant chemotherapy (NACT) has been developed as an alternative for PDS.

Two randomized trials (EORTC 55971 and CHORUS) have shown noninferior prognoses with a lower risk for postoperative adverse events in NACT groups compared with that in PDS groups of advanced OC patients (3–5). However, these noninferior results of the NACT groups (compared to PDS groups) was not confirmed in the JCOG0602 trial (6). Although results demonstrating that NACT can equate to PDS are conflicting, it is well acknowledged that patients with complete resection at PDS have the best prognoses. Thus, the use of NACT has been proposed for OC patients with disseminated, unresectable disease. In addition, appropriate selection criteria are urgently required for treatment strategies for OC patients.

Since 2015, the specialized ovarian cancer unit at our institution has implemented a personalized surgical approach for the treatment of OC patients (7). We adopted two-tier predictive models for R0 resection; specifically, a preoperative clinic-radiological assessment for all OC patients and a laparoscopic assessment, when necessary. In this paper, the authors report on the updated data from the study, which compared the survival between PDS and NACT groups of enrolled OC patients per our selective protocol.

## Methods

### Data Collection

This study was conducted according to the Declaration of Helsinki and was approved by the Committee at Fudan University Shanghai Cancer Center. All of the individual participants consented to the use of their medical records for research purposes.

Between Sep 1^st^, 2015, and Aug 31^st^, 2017, 161 OC patients were enrolled in our prospective personalized treatment cohort. All of the patients had preoperative clinic-radiological assessments, according to the Suidan criteria for R0 resection (8). Patients with a score of 0–2 were determined to have PDS. Patients with a score of ≥3 were counseled on the choices of PDS, NACT, or further laparoscopic assessments, according to the Fagotti criteria (9).

The pathological diagnoses were reviewed (according to the WHO criteria) by two experienced gynecological pathologists. Additionally, the patients were staged according to the 2014 FIGO criteria. R0 was defined as the absence of macroscopic residual disease (RD) after surgery. According to the response to the platinum-based chemotherapy, patients were classified as being either platinum-sensitive or platinum-resistant (10, 11). Progression-free survival (PFS) was defined as the interval ranging from the date of the primary surgery or the first cycle of neoadjuvant chemotherapy to the date of disease progression or recurrence. Overall survival (OS) was defined as the interval ranging from the date of the primary surgery or the first cycle of neoadjuvant chemotherapy to the date of death or the last follow-up (May 1^st^, 2020).

### Statistical Analyses

SPSS statistical software (version 26.0, SPSS, IBM Inc., New York, USA) was used for the statistical analyses. Descriptive statistics were used for the demographic data, and the data were summarized as medians with ranges or frequencies with percentages. The PFS and OS were analyzed with the Kaplan-Meier method and with log-rank tests in the univariate analyses. For the multivariate analyses, a Cox regression analysis was used to evaluate the effects of the prognostic factors, which were expressed as hazard ratios (HRs). P<0.05 was considered to be statistically significant, and all of the reported P values were 2-sided.

## Results

### Patient Characteristics

The patient characteristics are summarized in **Table 1**. The median (range) age was 57 (27–77)-years-old. Among the 161 OC patients who were enrolled, 110 patients underwent PDS, and 51 received NACT, with corresponding interval debulking surgery (IDS). The majority (158/161, 98.1%) of the patients were of an advanced FIGO stage, including 128 patients with stage III disease and 30 patients with stage IV disease. The R0 resection rate was 57.8% in the whole cohort. Detailed residual disease data were shown previously. That is, 62 (56.4%) patients in the PDS group had no residual disease, while 31 (28.2%) and 17 (15.5%) patients received R1 and R2 resections, respectively. In the NACT group, 60.8% (31/51) of patients had R0 resection, while the R1 and R2 rates were 29.4% (15/51) and 9.8% (5/51), respectively. All but one of the patients received platinum-based chemotherapy, and 105 (65.2%) patients were platinum-sensitive. Besides, 91 patients in our cohort had germline *BRCA* mutation tests. Among them, 31 patients harbored deleterious *BRCA* mutations.

**Table 1:** Patient characteristics (n = 161).

Age	Median (range)	57 (27–77)	
**FIGO Stage**	Early	3	1.9%
	Advanced	158	98.1%
	Stage III	128	79.5%
	Stage IV	30	18.6%
**Family history**	Yes	54	33.5%
	No	107	66.5%
**Treatment strategy**	PDS	110	68.3%
	NACT+IDS	51	31.7%
**Residual disease**	R0	93	57.80%
	RD	68	42.20%
**Platinum sensitivity**	Yes	105	65.2%
	No	48	29.8%
	NA	8	5.0%
**Status**	Alive	108	67.10%
	Dead	50	31.10%
	Censored	3	1.90%

### Prognostic Impacts of the Different Treatment Strategies

**Figure 1:**
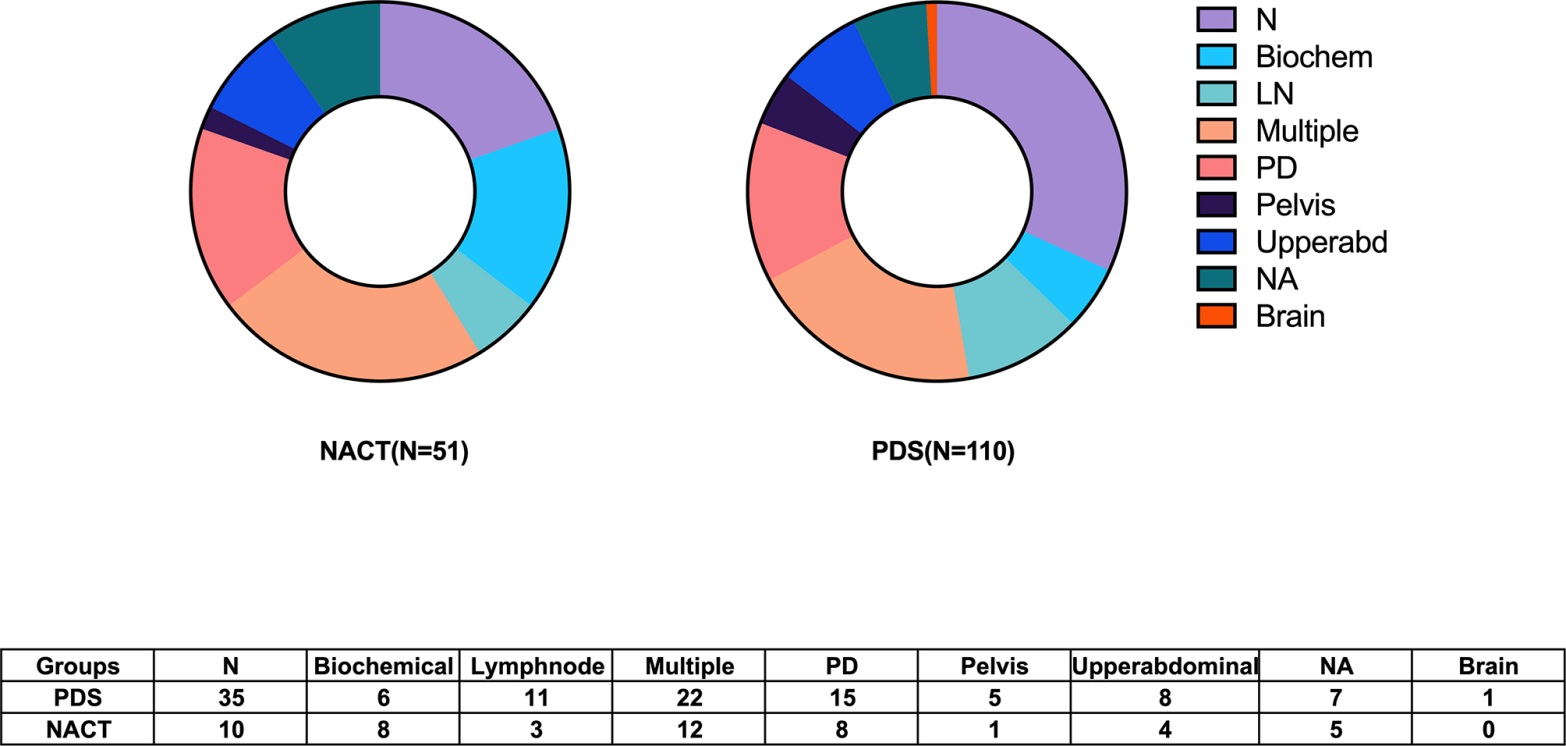
Recurrence patterns of disease between the NACT and PDS groups.

The median follow-up time was 38 (1–53) months. The median (95% confidence interval, CI) PFS was 18 (14.6–21.3) months. Six patients in the PDS group died within half a year. Among them, four patients received R0 resections, and died of severe perioperative complications. While another two patients had bulky residual disease, and died of disease progression. Furthermore, the histological types of two patients indicated clear cell cancer. Twenty-two (13.7%) women experienced disease progression during adjuvant chemotherapy, and eighty-seven (54.0%) patients exhibited documented recurrence. The recurrence patterns between the two groups are shown in **Figure 1**. Eleven patients had secondary cytoreductive surgery with R0 resection. Eight platinum-sensitive recurrent patients had PARP inhibitor maintenance, including six with niraparib and two with olaparib. The median OS was not able to be estimated. One hundred and eight (67.1%) patients were still alive at the time of the last follow-up, and fifty (31.1%) deaths were documented.

The known negative effects of platinum resistance on PFS (P<0.001) and OS (P<0.001) were confirmed. Although patients with clinic-radiological scores of 0–2 had prolonged PFS and OS, compared to patients with a score of ≥3, the differences were not significant (P=0.147 and P=0.441, respectively).

**Figure 2:**
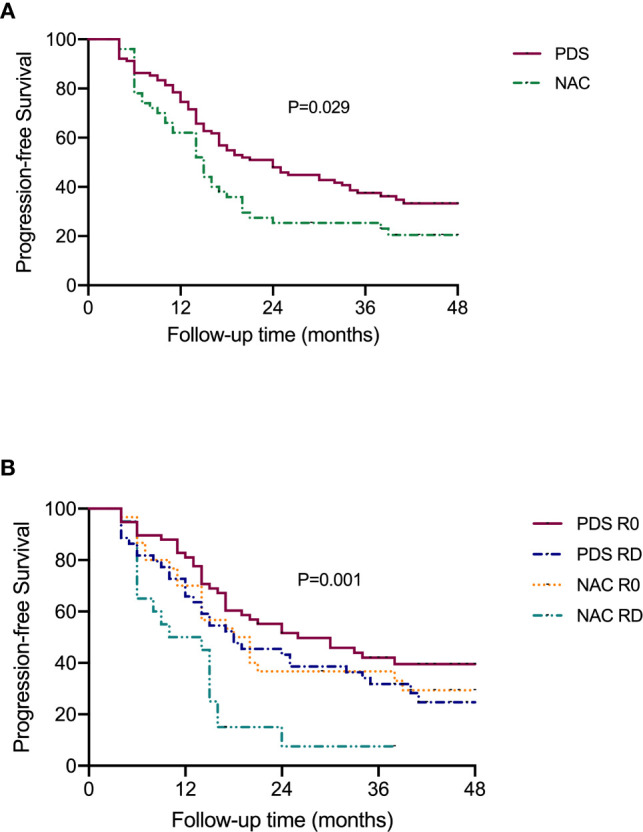
Progression-free survival of patients according to the subgroups. **(A)** Stratified by different treatment strategies. **(B)** Stratified by treatment strategies and residual disease.

**Figure 2** shows the PFS according to the treatment arm and debulking results. Based on the univariate analysis, it was demonstrated that the PDS group had prolonged PFS compared with the NACT group, with median (95% CI) PFS values of 24.0 (16.7–31.3) months and 15.0 (11.9–18.1) months, respectively (P=0.029, **Figure 2A**). The median (95% CI) PFS values for patients with R0 resection at PDS, residual disease at PDS, R0 resection at IDS and residual disease at IDS were 26.0 (12.1–39.9) months, 18.0 (8.7–27.3) months, 18.0 (11.6–24.4) months, and 10.0 (0–20.96) months, respectively. The subgroup analysis showed that patients with residual disease who received NACT had the worst PFS (P=0.001, **Figure 2B**). Based on the multivariate analysis, it was demonstrated that NACT with residual disease was still an independent impaired factor for PFS [HR=2.011 (1.031–3.923), P=0.04, **Table 2**].

**Table 2:** Cox regression analysis of PFS.

Characteristics		OS				
		HR	95% CI			P value
**Age (continuous variable)**		0.970	0.950	–	0.990	0.004
**Platinum sensitivity**	**No**	Referent				
	**Yes**	0.014	0.006	–	0.031	<0.001
**Patterns of residual disease**	**PDS with R0 resection**	Referent				
	**PDS with RD**	1.448	0.882		2.377	0.143
	**NACT with R0 resection**	1.209	0.666	–	2.194	0.532
	**NACT with RD**	2.011	1.031	–	3.923	0.040
**Clinic-radiological score**	**0**–**2**	Referent				
	**≥3**	0.575	0.346	–	0.955	0.033

**Figure 3:**
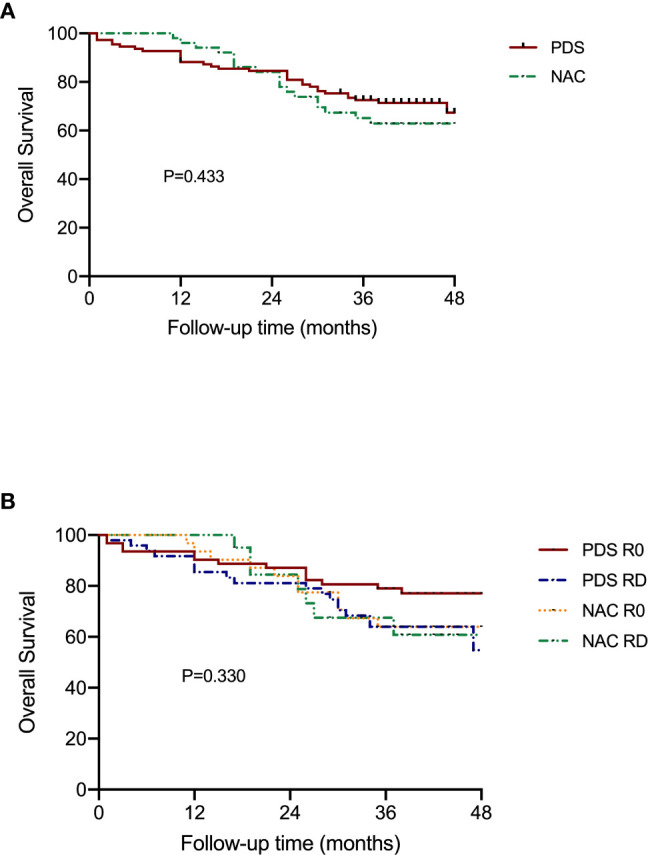
Overall survival of patients according to the subgroups. **(A)** Stratified by different treatment strategies. **(B)** Stratified by treatment strategies and residual disease.

**Figure 3** shows the OS according to the treatment arm and the residual disease status. Based on the univariate analyses, it was demonstrated that neither the different treatment strategy nor the residual disease status affected OS (P=0.433 and P=0.330, respectively). Additionally, there was no difference among the groups that were stratified by residual disease and treatment strategy subclassification (**Table 3**).

**Table 3:** Cox regression analysis of OS.

Characteristics		OS				
		HR	95% CI			P value
**Age (continuous variable)**		0.970	0.940	–	1.000	0.051
**Platinum sensitivity**	**No**	Referent				
	**Yes**	0.049	0.022	–	0.108	<0.001
**Patterns of residual disease**	**PDS with R0 resection**	Referent				
	**PDS with RD**	1.769	0.787		3.977	0.167
	**NACT with R0 resection**	2.054	0.806	–	5.231	0.131
	**NACT with RD**	0.663	0.237	–	1.858	0.435
**Clinic-radiological score**	**0**–**2**	Referent				
	**≥3**	0.710	0.323	–	1.562	0.395

## Discussion

In our prospective cohort, we validated two-tier predictive models for R0 resection to determine if OC patients were to receive PDS or NACT. After a greater than three-year follow-up period, we demonstrated that NACT ovarian patients had inferior PFS, when compared with PDS patients. However, there was no difference in the OS between the two groups.

As we have previously reported, our two-tier predictive algorithm is convenient for OC treatment decision-making (7). The R0 rate of debulking surgery has been improved to 57.8% compared with the rate of 31% based on our historical data (10). Additionally, these results are comparable to those from other studies, which have reported R0 rates ranging from 41% to 67.8% (11–13). Given our prospective feature, the data from our personalized cohort were objective and reliable.

The choice of NACT as an effective alternative for PDS of OC treatment has been debated for years. Two randomized trials (EORTC 55971 and CHORUS) have reported that patients in the NACT group achieved higher R0 rates and lower perioperative complication rates, when compared to those patients in the PDS group (3, 5). Furthermore, there were no survival differences between the two groups. However, the R0 rates in the PDS groups were below 20%, which were much lower compared with the rates in other studies (14, 15), and it is not clear whether maximal efforts were performed during PDS. Thus, several scholars have challenged the concept of substituting NACT for PDS. Onda et al. (6) have reported of another noninferior phase III randomized trial to compare PDS and NACT in OC treatment. In this study, a survival noninferiority effect of NACT was not confirmed (compared with PDS), with median OS values of 44.3 and 49.0 months, respectively.

In our cohort, the patients in the NACT groups exhibited impaired PFS, when compared to those patients in the PDS group, whereas there were no survival differences from the OS analysis. In contrast from the previously mentioned randomized trials, our prospective cohort demonstrated a validated two-tier algorithm for treatment strategy determination. As a result, the patients with a higher tumor burden tended to be allocated to the NACT group. Thus, we used the clinic-radiological score as an indicator for tumor burden in the multivariate analyses. It was demonstrated that NACT with residual disease was still an independent impaired factor. In regard to the OS analysis, although nearly five years had passed since the first patient’s enrollment, 67.1% patients were still currently alive, and the median OS was not able to be estimated. Thus, further follow-ups are needed.

Furthermore, it should be noted that six patients in the PDS group died within half a year. Among them, four patients had R0 resections with extreme debulking surgery and perioperative complications. Similar to the results of the SCORPION trial, over half of the OC patients with a high tumor burden who received PDS experienced perioperative moderate or severe morbidities (16, 17). Patients with high tumor load had worst prognosis, while there was no prognostic difference between PDS and NACT groups for this subgroup (18, 19). Narasimhulu et al. (20). demonstrated the use of the Mayo triage algorithm to identify OC patients who were at the highest risk of morbidity and mortality after debulking surgery. They included indicators such as albumin, age, and presumed surgical complexity to determine the uses of either upfront surgery or chemotherapy. Thus, the decision on whether to use PDS or NACT for OC patients should consider not only the resectability of the tumor burden but also the tolerability of the patients. NACT could be a candidate for OC patients who cannot afford extensive surgical procedures to achieve a complete cytoreduction.

In addition, 11 patients received secondary cytoreductive surgeries with R0 resection. The recent data of Desktop III have shown as surgery resection can improve the prognosis. Marchetti C et al. (21) also reported the benefit of secondary cytoreduction even in *BRCA* mutated patients with olaparib maintenance. Besides, eight platinum-sensitive recurrent patients had PARP inhibitor maintenance. And these above might also influence the overall survival analysis.

In conclusion, NACT ovarian patients exhibited inferior PFS compared with PDS patients in our prospective cohort. In addition, the OS data require further follow-ups. Given the nature of our selective protocol, NACT cannot be arbitrarily denied while appropriate PDS is still a priority.

## Data Availability Statement

The raw data supporting the conclusions of this article will be made available by the authors, without undue reservation.

## Ethics Statement

The studies involving human participants were reviewed and approved by the Committee at Fudan University Shanghai Cancer Center. The patients/participants provided their written informed consent to participate in this study.

## Author Contributions

ZF and HW participated in the study design, carried out the data collection, performed the statistical analysis, and drafted the manuscript. RL, SL, and YF carried out the data collection and participated in the radiological review. RB participated in the pathologic review of all slides. XC carried out the data collection. XJ and XW conceived the study and participated in its design and coordination. All authors read and approved the final manuscript. All authors contributed to the article and approved the submitted version.

## Funding

This work was financially supported through a grant from the leading project of the Science and Technology Commission of Shanghai Municipality (No. 19411960200) for XW.

## Conflict of Interest

The authors declare that the research was conducted in the absence of any commercial or financial relationships that could be construed as a potential conflict of interest.
